# The use of 360-videos to bridge the gap between pre-clinical and clinical dental teaching

**DOI:** 10.1186/s12909-025-06946-w

**Published:** 2025-03-13

**Authors:** Nabeela Caratela, Menna Shykhon, Michael Milward, Zehra Yonel

**Affiliations:** 1 Birmingham Dental Hospital, Birmingham, England, UK; 2 Periodontology Research Group (PRG), Department of Dentistry, School of Health Sciences College of Medicine and Health, Birmingham Dental Hospital and School, Birmingham, England, UK; 3 School of Dentistry, College of Medical & Dental Sciences (MDS), Institute of Clinical Sciences (ICS), University of Birmingham, Birmingham, UK

**Keywords:** Dental Education, Focus Groups, 360-Videos

## Abstract

**Background:**

The transition from pre-clinical to clinical teaching is often a time of heightened anxiety for students. With the shift to bi-modal teaching during the pandemic there was an opportunity to explore the use of 360-degree videos and virtual reality (VR) simulation teaching to enhance educational experience and smooth the transition from pre-clinical to clinical teaching. The aims of this study were to understand students’ perceptions of face-to-face and virtual simulation teaching during the recovery phase of the COVID-19 pandemic.

**Methods:**

Two groups of students were recruited all of whom were about to have their clinical introduction to the periodontology department. All 20 students received current standard induction programme. One group (*n* = 7 students) received standard teaching only. One group (*n* = 13 students) in addition to standard teaching methods also received access to 360-degree video and VR headset prior to standard teaching. Focus groups were then conducted with the students. A topic guide was developed and piloted. Focus groups were conducted online, audio was recorded and transcribed verbatim. Transcripts were analysed codes and themes were developed using thematic analysis as a framework for analysing the focus groups.

**Results:**

The 3 key themes identified were: the importance of familiarity with the clinical environment, preparation prior to attending clinical sessions and the benefit of practical experience.

**Conclusion:**

This study demonstrates how 360-videos and VR technology may enhance dental education, provided it is implemented appropriately and at the correct time in training. Overall, students had a positive attitude towards using 360-videos and acknowledged its value in meeting a range of learning objectives, including infection control, IT training, and clinic orientation.

**Supplementary Information:**

The online version contains supplementary material available at 10.1186/s12909-025-06946-w.

## Introduction

The COVID-19 pandemic posed global challenges to the delivery of dental care and dental education. The provision of dental education needed to rapidly adapt with a move away from traditional face-to-face didactic teaching methods to the use of virtual learning methods as lockdown measures forced universities to pause in-person clinical training. Bi-modal learning is the process of acquiring knowledge using two types of content at the same time. In this case, it refers to online lectures supplementing face to face learning. This shift from traditional methods to bi-modal learning posed many challenges but also conferred several benefits regarding opportunities to enhance and update delivery of educational content [1, 2].

The transition from pre-clinical to clinical training has often been highlighted by dental undergraduates as a particularly daunting period [3, 4]. Dental schools have facilitated this transition through a gradual introduction to the clinical environment, starting with clinical inductions, shadowing peers and qualified staff members and lectures and tutorials outlining expectations in the clinical environment. Students often cite nervousness, apprehension and anxiety prior to commencing the clinical phase of training [3, 4]. These emotions were compounded when students returned to in-person teaching, due to the lack of exposure to the clinical learning environment during the COVID-19 pandemic as students were taught exclusively online [1, 2]. To address these challenges and enhance students’ preparedness for clinical practice, novel approaches such as 360-videos have been explored and implemented in medical and dental education to bridge the gap between theoretical knowledge and practical clinical experience [5].

Virtual reality (VR) uses digital technology to simulate real-life environments or situations in a three-dimensional digital interactive platform [5]. The use of virtual reality in medical and dental education in increasing and complements the existing traditional learning methods [6, 7]. 360-videos are immersive video recordings where the environment is recorded using a 360-camera; the user can wear a headset, allowing them to view and interact with the environment. The user can control and alter their view through head movements, allowing them to feel present in the space. The aim of 360-videos is to provide a safe environment where students can develop their skills and familiarise themselves with the clinical environment and equipment routinely used without external pressures. The use of 360-videos has shown to improve learner experiences by providing real-life scenario simulations, stimulating emotional engagement and motivation through interaction, enabling development of empathy by experiencing patient perspectives and improving independence via remote learning opportunities [8]. As 360-videos are a relatively new development, evidence regarding their use as an educational tool is disparate, suggesting further research is required to explore the learner’s experience and effectiveness of the technology [8, 9].

Given the growing use of 360-videos and virtual reality in medical education, it was felt its application in the pre-clinical phase of dental education could mitigate some of the challenges and negative emotions reported by students. As such, a 360° immersive virtual reality video was designed that showed the clinical environment. This video was compatible with a virtual reality headset and contained interactive buttons which allowed students to immerse themselves in the clinical environment. The interactive buttons were strategically placed on key items / resources in the clinic to signpost students to additional learning resources, including videos, podcasts, documents and standard operating procedures.

This study therefore aimed to understand students’ perceptions of face-to-face and virtual simulation teaching during the recovery phase of the COVID-19 pandemic, as in-person teaching resumed. Focus groups were conducted, with groups of undergraduate students to learn which aspects of education delivery they found of greatest benefit. The results of this study will inform development of educational resources for the future dental workforce.

## Methodology

This study complies with the Helsinki Declaration and ethical approval was granted (ERN_2022-0577) to conduct focus groups with undergraduate dental students at the University of Birmingham, School of Dentistry, to understand their views regarding educational methods for improving the transition from pre-clinical to clinical phases of dental training.

The inclusion criteria of the study were:

1. Voluntary participation in the study
2. Being in the transition period of pre-clinical and clinical teaching [Periodontology]
3. Completion of the full periodontology standard induction programme

To develop the 360-video, the 360-degree camera, ‘insta360 Pro 2’, was used to capture the images of the clinical environment. This was then used in conjunction with ‘ThingLink’, a program that enables annotation of digital media to develop the 360 images. “Buttons” were added to key structures (e.g. the dental chair), and when these “buttons” were clicked, the video linked-out to further information. This additional information was sometimes written text/audio description, other videos, key documents, standard operating procedures or audio files.

A participant information sheet relating to the research study went out to students on the Bachelor of Dental Surgery Undergraduate programme at the University of Birmingham, who were about to embark on the periodontology clinical training. By this stage in their undergraduate course, students had completed a simulation course in a clinical skills lab prior to starting clinical work on patients. They would have 1 year experience on a general “dental clinical practice” clinic, and 4 months after commencing this, would have started additional specialty clinics in Periodontics, Prosthodontics and Conservative dentistry. Although the length of time spent on clinics would be the same, there may be variation with the students’ clinical experience and cases seen by this point.

A total of 20 students meeting the eligibility criteria volunteered to participate and completed the written study consent form.

The participants were allocated to one of two groups. The first received standard teaching as per the programmed curriculum. The intervention group received the same standardised programme of teaching delivered by the same member of staff. Additionally, the intervention group were given access to an online 360-video and associated resources, as well as a virtual reality headset two-weeks prior to attending the standardised clinical induction. The 360-video of the clinical environment was embedded on a page within the online learning platform, ‘Canvas’, used by the University of Birmingham. Access to this was limited to the students in the intervention group. Students could navigate around the video and click on the relevant “buttons” which provided links to additional resources. Each student was also given a VR headset which they could use in conjunction with their mobile phone to utilise the 360-video as an immersive VR educational tool, if they desired to do so.

A topic guide (see Supplementary material) to facilitate focus groups was developed and piloted. Three focus groups were then undertaken with the 20 students to understand their thoughts and feelings regarding periodontology induction experiences. Each focus group consisted of 5–8 students. Focus groups were conducted by the facilitator ZY via Zoom and recorded. The facilitator was a clinician from the Periodontology specialty and related the use of VR technology to how this can help students during the induction onto the Periodontology clinic. A semi-structured questioning process was undertaken to ensure that participants spoke without interruption on each question with the use of open and closed questions and follow-up probes. Participants also had the opportunity to add anything related to the questions asked or related to the overall research topic at the end of the focus group.

The audio files were transcribed verbatim by the authors NC and MS, and checked by the project lead, ZY. The anonymised transcripts were imported into NVivo, a qualitative data management software application, and the data was analysed using thematic analysis [10]. The transcripts were read multiple times to become fully immersed with the content and to identify the core ideas in the data, and to develop a coding framework. Data was coded via an inductive approach to identify patterns which were then grouped into themes using an iterative process. NVivo software application was used to code the transcripts. In-person meetings were organised to discuss similarities and differences in the themes and interpretations, and a consensus was achieved. The proposed coding was discussed and agreed upon with the project lead.

## Results

The focus groups lasted between 45 and 60 min. The facilitator led the focus groups to ensure key topics were considered but students were given the opportunity to discuss topics outside of this and an open and honest conversation environment was promoted.

In total, 20 students participated in the research study. 11 identified as female, 9 identified as Male. Seven Students were exposed only to traditional teaching methods, 13 students had access to the enhanced educational resources 2-weeks prior to receiving standardised teaching.

The key themes from the thematic analysis included: importance of familiarity with clinical environments; preparation prior to attending clinical sessions and the benefits of practical experience.

### Theme 1: importance of familiarity with clinical environment

Student felt that introduction to clinics and familiarity with staff members and the clinical environment is important. It helped to relieve anxieties when starting new clinics and provided “reassurance.” Students also felt that sessions early on in their preclinical years where they assisted older students helped to improve familiarity with the clinical environment. Where student hadn’t already been exposed to clinics, they felt the use of the VR headsets would be very useful as it gave an idea of the layout of the clinic and where to find instruments or materials. However, once they had already been exposed to clinics they felt it didn’t offer a great deal extra.

“I think you could have a video also in the dirty room and the clean room, because that’s quite an important part of clinic like you go into those areas every time you enter clinic. And I just think it’s good having a little demo as well in there about what you do with trays, supps, the hand pieces.”

Students believe that consistency within teaching was important and found that different clinicians may have different techniques, and this could cause confusion when starting out on clinics. This could be mitigated with the use of standardised teaching materials. Having the same supervising clinicians made students feel more at ease and felt it was important for clinicians to know their capabilities. Smaller group tutorials are preferred for students who felt they were more “personal” and felt less anxious to contribute to discussions.

### Theme 2: preparation prior to attending clinical session

Students felt it was important to be prepared prior to attending tutorials and clinical sessions. They felt preparation helped to relieve anxiety and allowed students to know “what [they’re] going to face in the session.” Students also felt it was valuable to set expectations prior to tutorials so they are aware what is expected from them.

“I personally find having resources in advance extremely useful. I find that it helps me plan exactly what I need to do for that session beforehand.”

“Because we didn’t know what the session was going to be on, I was nervous because I hadn’t done much preparation for that session… I think just the not knowing…caused some anxiety”

A range of formats was suggested including handbooks, videos and the use of VR headsets was also discussed. Traditional handbooks were deemed to not be useful as they contained too much information and were “overwhelming.”

“Sometimes when you get loads of information all at once, it’s kind of hard to absorb.”

Watching videos before a tutorial, followed by a live demonstration can allow students to see procedures form a different perspective and “change the way you view things.”

Students felt that the VR headset was a “cool concept” but the headset itself provided little over the 360 video. Although, it should be highlighted that the headset provided did not have controllers and incorporating this feature may allow for a more interactive experience.

The importance of the accessibility of this information was highlighted. Students wanted this information to be easily accessible and concise. Students felt information was more useful when presented in “bite sized” amounts or on a “week by week” basis. Emails and “WhatsApp” groups were considered more accessible ways of sharing information and can be easily viewed on mobile phones. They would also like access to this information to refer to after these sessions and felt investment into resources was “worth it for the long-term future” use.

### Theme 3: benefits of practical experience

Students favoured practical experience over traditional lecture-based tutorials which were deemed “less engaging.” They wanted the opportunity to practice clinical techniques whilst being able to gain feedback on their performance. Mock patients were suggested as an idea to help students prior to treating patients:

“Mock patients is an interesting idea because it simulates exactly what you’ll be doing on clinic, just without the pressure of patient expectations.”

“I think hands on experience is the best way, but without the if you mess something up, this has consequences on a real patient. If it’s on simulation, then I think that’s perfect.”

It was recognised that such exercises consumed significant time and resources, but this could be a useful application of VR headsets. Some felt that videos were less interactive than live demonstrations and that information “doesn’t really sink in until you do it yourself.”

“In person demos are so much more helpful than watching videos.”

An immersive experience such as VR headsets may be more engaging and allow students to retain more information.

“I think, perhaps maybe having videos for specific procedures. Imagine putting on the headset, and you were there in a bay, and it was a clinician, and they were showing you how to do a buccal infiltration. I think that would be quite cool, or just like setting up a rubber dam or something like that.”

The importance of the timing of these exercises was highlighted. Student felt this would be useful prior to starting clinics in the preclinical years. However, once they had already started treating patients, these exercises were less valuable.

The use of computer software systems was another area where students struggled and caused additional anxieties and was “intimidating” when starting out on clinics:

“I feel like that makes you the most nervous because that’s when the patients watching you and you can’t, you know, they’re looking at what you’re writing. And if you can’t work a system, it can be quite embarrassing”

**Fig. 1:**
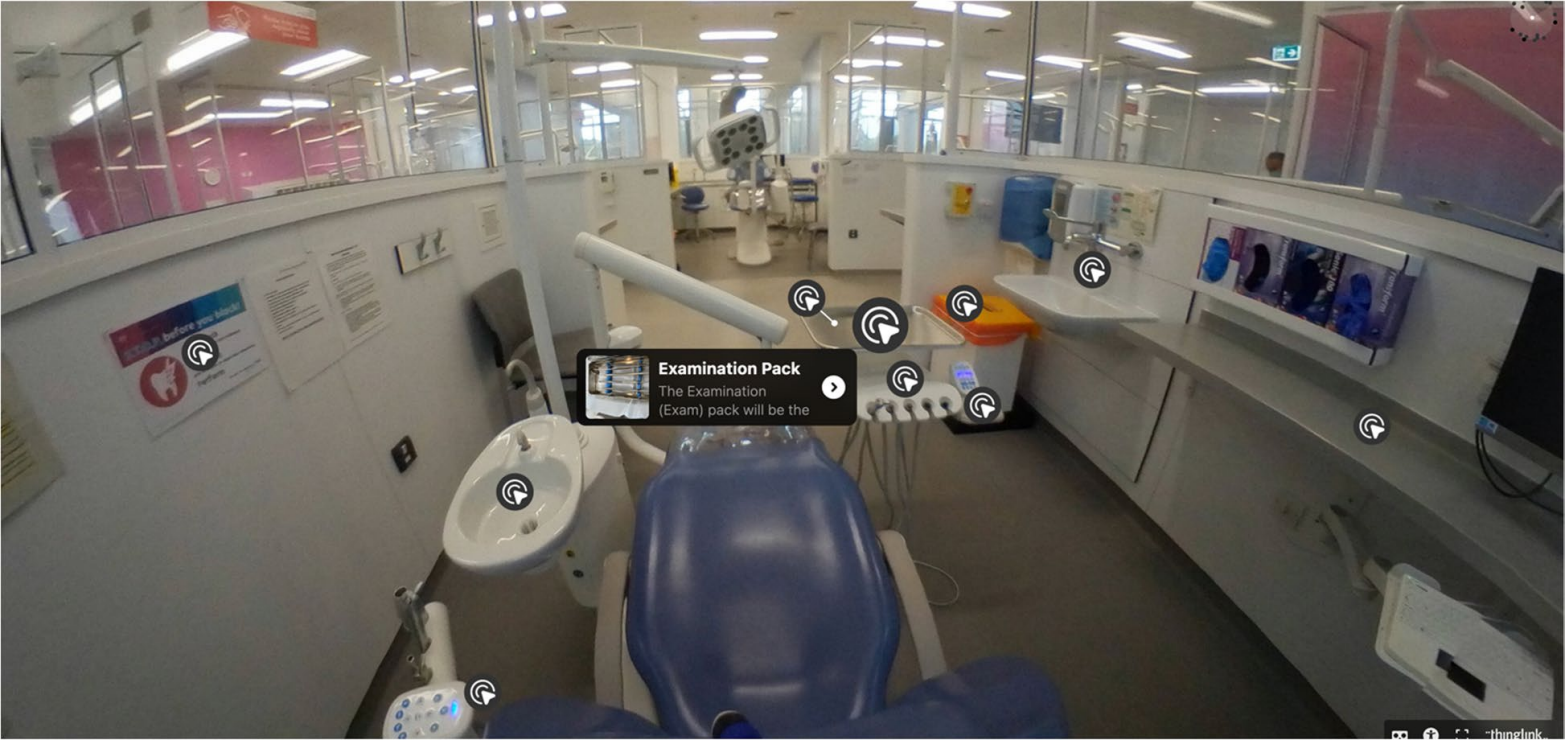
Screenshot from interactive video which was used alongside VR headset demonstrating the clinical set up

There are a range of different computer software systems used including educational platforms and clinical software for note taking, radiography, booking systems and clinical photography. Students felt that simulations that allowed them to familiarise themselves with these computer systems would be beneficial and could be a further application of the use of VR headsets. Again, the importance of the timing of training was stressed and it was highlighted that this should be provided close to the time where these skills would be applied on clinics (Tables 1, 2, 3 and 4, Fig. 1).

**Table 1:** Participant information

Female: Male	11:9
Standard Teaching only Group	7
Standard Teaching + immersive 360-Video	13

**Table 2:** Example of most common codes

Familiarity on clinics	Preparation
Anxiety	Practical Experience
Mock scenarios	Key information
Training timing	Clinical assist
Orientation	Small groups
IT skills	Ease of access
Expectations	Session plan
Reassuring	Unprepared

**Table 3:** Themes

Theme 1	Importance of familiarity with clinical environment
Theme 2	Preparation prior to attending clinical session
Theme 3	Benefit of practical experience

**Table 4:** Key quotes from participants in support of the themes in [] interpretation and context provided to support the quote

“I think you could have a video also in the dirty room and the clean room, because that’s quite an important part of clinic like you go into those areas every time you enter clinic. And I just think it’s good having a little demo as well in there about what you do with trays, supps, the hand pieces.” [This participant provided ideas for additional 360 videos to support the transition from pre-clinical to clinical years. The suggestion here refers to infection prevention control]	“I personally find having resources in advance extremely useful. I find that it helps me plan exactly what I need to do for that session beforehand.” [Here the participant is explaining how having access to the 360 videos as an aid to preparation to clinics was useful to help plan for clinical sessions.]
“Because we didn’t know what the session was going to be on, I was nervous because I hadn’t done much preparation for that session… I think just the not knowing…caused some anxiety” [This participant acknowledged that the unknown and anticipation of a new environment, with new staff in a new clinical setting is a source of anxiety. Knowing in advance and being able to watch a 360 video may reduce anxiety]	“Sometimes when you get loads of information all at once, it’s kind of hard to absorb.” [This participant highlighted that during the standard teaching induction a lot of information is given over an entire morning with lots of information given at once on a broad range of topics (clinical / administrative/ staff introductions / locations of equipment and resources). This can be overwhelming, and the quantity of information may be difficult to take on board. Thus, supporting the concept of providing a resource to refer back to such as the 360 video]
“Mock patients is an interesting idea because it simulates exactly what you’ll be doing on clinic, just without the pressure of patient expectations.” [This participant felt the 360 video with mock patient interactions may remove pressure and enhance learning]	“I think hands on experience is the best way, but without the if you mess something up, this has consequences on a real patient. If it’s on simulation, then I think that’s perfect.” [This participant suggested face-to-face / practical exposure is ideal, however 360 videos provide a safe alternative with lower stakes and less risk which may enhance learning]
“In person demos are so much more helpful than watching videos.” [This participant expressed a preference for more in-patient session than videos]	“I think, perhaps maybe having videos for specific procedures. Imagine putting on the headset, and you were there in a bay and it was a clinician, and they were showing you how to do a buccal infiltration. I think that would be quite cool, or just like setting up a rubber dam or something like that.” [This participant suggested further videos which may support learning including some examples of practical and clinical procedures]
“I feel like that makes you the most nervous because that’s when the patients watching you and you can’t, you know, they’re looking at what you’re writing. And if you can’t work a system, it can be quite embarrassing” [This participant was recounting how computer software systems can be daunting and when trying to navigate the systems in view of the patient if you are not efficient it can be a source of embarrassment. They went in to suggest a 360 video demonstrating use of administrative systems allowing practice in a simulated environment can be reassuring and reduce potential embarrassment.]	“I think that it would be very useful for people who have like never really been on clinic to see that.” [This participant explains how 360 videos introducing the department may be useful for pre-clinical students who have not familiarised themselves to the clinical setting, but such videos are less useful after students have started on the clinic. Thus, the timing students are exposed to these resources is key for them to have most impact.]
“I agree with Student 9 in terms of that, it [360 video] could be useful. However, I think the whole idea of getting the headset and setting it up and putting in the phone, downloading an app. It’s a lot of effort that students may not want to do… the headset, I think it’s a lot more effort to set up, that might put students off, and you might as well just watch the 360 video” [This participant described the challenges of the VR headset and how the 360 video alone may be better than the video + headset]	“I feel like it wasn’t the best use of the VR headset. Like the other student said, you could just watch it (the 360 videos) on your phone and click through it. And that was still a nice concept and a nice idea. But I think, in terms of the expense of the VR headset and the expense of setting them up, it could be used for other things [This participant identified barriers to the VR headset but felt the 360 video was of benefit]

## Discussion

### Key findings

#### Familiarity with clinical environment

The application of clinical knowledge and skills in a real clinical scenario is a key objective of dental education. Students expressed stress and anxiety associated with transitioning from the pre-clinical to clinical stage of training, and these feelings have also been expressed by students in medical training [11]. As new clinical elements are introduced in training, students may feel unprepared and incompetent. This study identified the potential for VR technology to be used as a clinical orientation tool, facilitating students’ familiarity with the clinical environment prior to starting on clinics. The use of VR is not limited to simulating clinical procedures but can also be used to expose students to peripheral skills such as infection control protocols and locating and setting up dental equipment. Familiarity with the clinical environment is paramount for students’ experiences. The availability of clinical space can also be a limiting factor to increasing clinical exposure. Utilising VR technology may help to reduce clinical induction times and increase clinical chair availability, whilst ensuring students are better prepared for clinics and thereby optimising their clinical exposure.

#### Preparation prior to attending clinical sessions

Students felt that preparation prior to clinical sessions was important, alongside an idea of clear expectations to reduce feelings of anxiety. When discussing preparatory resources, students found handbooks overwhelming due to information overload and saw value in the delivery of ‘bite-sized’, ‘week-by-week’ preparatory materials to be delivered via email or WhatsApp groups as an alternative. Furthermore, the students saw benefit in a bi-modal approach to education, suggesting videos of clinical procedures being made available prior to live clinical demonstrations. Evidence shows that using different teaching modalities can improve knowledge retention and accommodate different learning styles [12].

#### Benefits of practical experience

Students saw VR as an opportunity to gain practical experience via simulated scenarios. VR offers a safe environment, away from patient expectations and consequences of clinical errors [13]. The timing of the delivery of the resources was highlighted as an important factor, with students feeling that these resources would be most valuable if accessed prior to clinical exposure. If introduced at the correct stage in training, the findings suggest the benefits of VR in improving student confidence in clinics, reducing feelings of apprehension and therefore improve the transition to clinical training. These benefits of VR are not limited to dental education, and improved confidence in the ability to reproduce procedural steps has been demonstrated in surgical training when supplemented with VR [14]. Videos are also an easy adjunct to clinical education, however VR showed greater improvements in trainee confidence compared to video learning alone [14].

Furthermore, students expressed a desire to be involved in the process of implementing developing technology as they are best placed to inform on whether these advances are useful or not. Students are more likely to engage with novel changes when they are involved, rather than have it enforced upon them [15]. Students were keen to get involved with changes to the teaching methods and giving feedback to shape their learning experiences. They expressed an appreciation for being asked to give feedback by their educators and found incorporating technology into education interesting.

In this study, VR headsets were used for one specific application, however students suggested other possible uses, highlighting the versatility of VR headsets and their potential to address a wide range of student concerns including practical skills and IT-based skills. But timing is key, any 360-video be it operational, procedural, orientation based or instructional should come at the optimal time in training, as once the student has already experienced what the video is showing practically, the students deem the video content as redundant.

### Strengths of the study

With the emerging implementation of VR in medical and dental education, this study will undoubtedly contribute to the growing evidence base in this field, benefiting both educators and students.

This study focuses on a critical stage in dental education, the pre-clinical to clinical transition, therefore the findings of this study have the potential to have a significant improvement in the delivery of dental education.

Student feedback and engagement is highly important in the delivery of teaching, and this study reflects the importance of a student-centred approach to dental education, especially in the implementation of novel technologies. The use of focus groups in this study provides the opportunity for diverse groups of students to share their perspectives and insights in dynamic discussions guided by a trained facilitator; alternative data collection methods such as surveys and structured interviews may restrict the breadth of ideas curated.

### Weaknesses of the study

The small number of students (*n* = 20) who participated in this study were collectively from one single Bachelor Dental Surgery (BDS) programme; therefore the findings of this study may not be representative of undergraduates in other universities, or students from specific populations. Furthermore, the timing of introduction of clinical training varies between different BDS degree programmes, therefore student perceptions are likely to vary regarding the applications of VR. The results may not be transferrable to other institutions with different resources and cohort demographics.

Student participation in this study was voluntary, therefore increasing the risk of bias. Students who were interviewed are more likely to be engaged with the current undergraduate programme, have a particular interest in technology or have stronger opinions on the pre-clinical to clinical phase transition, therefore skewing the results. Furthermore, the focus groups were facilitated by ZY [author intials], who is an academic clinical lecturer on the BDS programme, hence participants may have felt hesitant to voice honest opinions despite efforts to ensure neutrality throughout. It is important to note the focus groups were conducted virtually, making non-verbal communication difficult to recognise which could have impacted the dynamic of the group conversations.

It is also important to highlight the limitations of 360-videos as an educational tool in comparison to fully immersive VR as highlighted in the Table 5. A fully immersive VR experience has the potential to be more versatile and interactive and may have yielded different results. This includes the potential to be beneficial even after the pre-clinical phase as it may offer the opportunity to enhance existing baseline clinical skills via real-life scenarios and decision-making.

**Table 5:** Comparison of learning elements in 360-video and fully immersive VR [16, 17]

	360-Videos	Fully Immersive VR
Interactivity	Limited to views captured by 3D camera	More interaction with the virtual environment and armamentarium.
Flexibility	Pre-recorded and fixed scenes	Adaptive with scenarios linked to operator actions.
Haptic Feedback	Not present	Haptic devices can be linked for tactile feedback.
Learning experience	Passive observation, with some interaction via ‘buttons’	Active learning with decision-making.

Moreover, this study focuses on subjective perceptions of 360 videos and VR in dental education and does not assess objective outcomes such as knowledge retention and assessment results. Consequently, any conclusions on the direct benefit of VR in dental education are tenuous.

## Conclusion

This study demonstrates how 360-videos and VR technology may enhance dental education, provided it is implemented appropriately. Overall, students had a positive attitude towards using VR and acknowledged its value in meeting a range of learning objectives, including infection control, IT training, and clinic orientation. Furthermore, students appreciated the possibility of VR being used to simulate clinical and patient encounters to provide holistic educational experiences prior to starting clinical practice. In the context of this study, it was felt that VR was not used to its full potential. Importantly, the feedback highlighted the importance of ensuring that VR complements and enhances existing teaching methods rather than being used as a novelty or for purely the purpose of introducing technology. The timing of its introduction is also critical, with preclinical years identified as the optimal timing for its integration to provide realistic learning opportunities before treating real-life patients. Students advocated for a balanced approach that combines VR technology with specific educational goals. The insights from this study provide a roadmap for refining the use of VR in dental education, informing educators of student views and preferences. The use of VR in medical and dental education is increasing; further research is necessary to explore student perceptions, effectiveness, limitations and applications of emerging technologies.

## Supplementary Information

Supplementary Material 1. Topic Guide for focus group.

## Data Availability

To access anonymised data contact the corresponding author. The anonymised qualitative data collected and analysed during the current study may be requested from the corresponding author.

## Abbreviation

VR: Virtual reality
