# Supplementary material for: The use of 360-videos to bridge the gap between pre-clinical and clinical dental teaching
Source: BMC Med Educ. 2025 Mar 13;25:375. doi: 10.1186/s12909-025-06946-w (PMC11905719; doi:10.1186/s12909-025-06946-w)
Supplement: Supplementary file 1 — Supplementary Material 1. Topic Guide for focus group. [file 12909_2025_6946_MOESM1_ESM.pdf]

Focus Group Topic Guide

The aim of this topic guide is to provide an overview to facilitate open and honest dialogue amongst the students involved in the research.

This topic guide has been informed by prior research, feedback from supervisors and PPI panel members. The topic guide will be flexible, it is important to ensure the views of students are heard. Thus, I want to avoid a prescriptive approach which may introduce biases through focusing on topics perceived by me to be of importance, which are perhaps less important to the students.

Thus, although broad and open questions will cover topics pertinent to the research question in the view of the research team. I am keen to hear the first-hand experiences of the students, so will use these broad topics to facilitate dialogue between students to gain insight into their views. The process will be iterative and will evolve as the research grows and each subsequent focus group may pick-up on themes and topics raised in prior focus groups if there is benefit in exploring these discussion points further. Additionally, at the start of the focus groups students will be informed that if there are any topics they want to discuss further or don't feel comfortable discussing in a group environment, or those who perhaps feel their voice wasn't heard will be given the opportunity to have an interview to ensure their views are understood.

<u>Introduction</u>

- Thank the students for agreeing to the focus group.
- Explain the format and estimated duration.
- Remind them attendance is voluntary, and they can choose to stop participating and leave the group at any time.
- Remind the participants the focus groups will be recorded and transcribed and thus to maintain anonymity I will not be using their names. Also, to try not to say anything that identifies themselves, their peers or other team members.
- At the start ask them to identify their study number for the audio tape to help when transcribing
  - i.e. I am a student, Study ID number 1. As sometimes it can be difficult to ensure a comment is attributed to the correct person.
- Reassure them if they do accidentally mention something identifiable it is not a problem as I can edit it out prior to transcription.
- Remind of the opportunity for supplementary interviews.
- Confirm they are happy to proceed

WP2
Version 0.1 Educational Enhancement – Focus Group Topic Guide

Nov 1st 2022

Broad Topics to cover with **ALL students** – with some potential additional questions if required, to help facilitate group discussion.

- How did you feel being involved in the research study process?
- Can you briefly explain your intro to Perio speciality teaching – what did it involve? how did it compare to other specialities? Did it fully meet your needs?
- What were your thoughts on the Introduction to Speciality teaching
- What were the challenges you experienced related to the introduction to speciality teaching?
- Were there any positives? If so, what?
- Do you feel you have the relevant skills / are you confident to start in the department?
- How did you feel **Before** having your introduction to speciality teaching?
- How did you feel **After** having your introduction to speciality teaching?
- Did you have any concerns prior to the induction which remain unaddressed?
- Are they any additional elements you would have liked to be covered which were not?

For those who had access to the video

- What were your thoughts on the video?
- Did you use it with or without the VR headset? What was your preference and why?
- Do you think there was benefit above standard teaching alone? If so, what?
- If you were to implement this going forward was there anything you felt worked well that you would keep the same?
    - Was there anything that didn't work that you would change?
- If you felt the videos were beneficial, where / how else would you like them to be developed. What other applications/ capacities would you find them beneficial?
- Are there any topics that you would like to discuss that I haven't mentioned?
